# Fewer hospitalizations and prolonged technique survival with home hemodialysis– a matched cohort study from the Swedish Renal Registry

**DOI:** 10.1186/s12882-019-1644-z

**Published:** 2019-12-30

**Authors:** Helena Rydell, Kerstin Ivarsson, Martin Almquist, Naomi Clyne, Mårten Segelmark

**Affiliations:** 1 Department of Clinical Sciences Lund, Nephrology, Lund University, Skane University Hospital, Njurmedicin exp A5:04, 171 76 Stockholm, Sweden; 2 Department of Clinical Sciences Lund, Surgery, Lund University, Skane University Hospital, Lund, Sweden; 3 Department of Nephrology and Department of Medical and Health Sciences, Linköping University, Linköping, Sweden

**Keywords:** Home hemodialysis, Institutional hemodialysis, Peritoneal dialysis, Hospital admission, Technique survival

## Abstract

**Background:**

Patients on home hemodialysis (HHD) exhibit superior survival compared with patients on institutional hemodialysis (IHD) and peritoneal dialysis (PD). There is a sparsity of reports comparing morbidity between HHD and IHD or PD and none in a European population. The aim of this study is to compare morbidity between modalities in a Swedish population.

**Methods:**

The Swedish Renal Registry was used to retrieve patients starting on HHD, IHD or PD. Patients were matched according to sex, age, comorbidity and start date. The Swedish Inpatient Registry was used to determine comorbidity before starting renal replacement therapy (RRT) and hospital admissions during RRT. Dialysis technique survival was compared between HHD and PD.

**Results:**

RRT was initiated with HHD for 152 patients; these were matched with 608 patients with IHD and 456 with PD. Patients with HHD had significantly lower annual admission rate and number of days in hospital. (median 1.7 admissions; 12 days) compared with IHD (2.2; 14) and PD (2.8; 20).

The annual admission rate was significantly lower for patients with HHD compared with IHD for cardiovascular diagnoses and compared with PD for infectious disease diagnoses. Dialysis technique survival was significantly longer with HHD compared with PD.

**Conclusions:**

Patients choosing HHD as initial RRT spend less time in hospital compared with patients on IHD and PD and they were more likely than PD patients, to remain on their initial modality. These advantages, in combination with better survival and higher likelihood of renal transplantation, are important incentives for promoting the use of HHD.

## Background

Morbidity and mortality remain high for patients on dialysis despite improvement during the twenty-first century [1–3]. Most earlier studies have shown better survival for patients on home hemodialysis (HHD), compared with patients on institutional hemodialysis (IHD) or peritoneal dialysis (PD) [4–11]. We corroborated these findings in earlier studies after matching for age and comorbidity [10, 12] and taking into account that patients with HHD, have a higher rate of renal transplantation compared with patients on IHD or PD.

Frequent and/or long-term hospitalizations severely impact a patient’s ability to live an independent life. Studying health care utilization, such as hospitalization provides us with an insight in the morbidity acquired during dialysis therapy. Dialysis technique failure is another important concern for patients on home-based dialysis modalities as it can cause temporary or permanent dependence on dialysis personnel with an unwanted switch to institutional hemodialysis.

There is a sparsity of reports comparing morbidity and hospital admissions between patients treated with HHD and IHD or PD and no previous study in a European population during the last decades [5, 6, 13–15].

The aim of the present study is to investigate morbidity in patients on HHD in comparison with patients on IHD and PD by analysing healthcare utilization and measuring time to first hospital admission, frequency and number of days in hospital, cause of hospital admissions and dialysis technique survival.

## Methods

### Criteria for definition of initial renal replacement therapy

HHD, IHD or PD as initial renal replacement treatment (RRT) were defined as the modality registered in the Swedish Renal Registry (SRR) at day 90 after start of RRT. Further criteria for definition of initial RRT are listed in Table 1.

**Table 1:** Definitions of HHD, IHD or PD as initial RRT, as based on modality day 90

	HHD		IHD		PD	
	Before day 90	After day 90	Before day 90	day 90–365	Before day 90	After day 90
Recovered renal function	Exclusion criteria	–	Exclusion criteria	–	Exclusion criteria	–
Renal transplantation	Exclusion criteria	–	Exclusion criteria	–	Exclusion criteria	–
HHD	–	–	Exclusion criteria	Exclusion criteria	Exclusion criteria	–
PD	Exclusion criteria	–	Exclusion criteria	Exclusion criteria	–	–

### Inclusion criteria

All adult patients (> 18 years) registered in SRR and starting renal replacement therapy between January 1st 1991 and December 31st 2012 were eligible for inclusion if they fulfilled the criteria of HHD, IHD or PD as initial RRT.

### Matching

The matching procedure has been described earlier in detail [12]. In short, each patient on HHD was matched with 4 patients on IHD and 3 patients on PD with the same sex, Charlson comorbidity index, age (+/− 3 years) and date of start of RRT (+/− 3 years). Charlson comorbidity index [16] was determined using all discharge diagnoses in the Swedish Inpatient Registry up to the start date of RRT as previously described [17]; this includes not only the ICD code of the main cause of the hospitalization but also of all co-morbidities.

### Collection of data

Dates of start and changes of RRT, dates of birth and renal diagnosis were collected from SRR. Discharge diagnoses and dates of hospital admissions were collected from the Swedish Inpatient Registry. Dates of death were collected from the Swedish Mortality Database.

### Comparisons of hospital admissions

The comparisons of hospital admissions between patients on HHD and patients on IHD or PD, respectively, were performed in three ways: as annual hospital admission rate, days admitted per year and time to first hospital admission.

Only admissions from day 90 after start of RRT were included in the analyses. The follow up was defined according to two different approaches. With follow up per protocol, only admissions that started while the patients were still on their initial RRT were included. In the analysis of time to admission, censoring was performed at change of RRT, death or end of study. With the intention to treat follow up, all admissions until end of the study, December 31st 2013, were included. In the comparisons of time to first admission, censoring was only performed at death or end of study.

### Admissions with cardiovascular and infectious disease diagnoses

Separate comparisons were performed for annual hospital admission rate and time to first admission for cardiovascular diagnoses or acute infectious disease diagnoses. The diagnoses used in the assignment of cardiovascular admissions and infectious admissions could be categorized as either principal or secondary diagnoses. Albeit, only a few acute cardiovascular diagnoses categorized as secondary diagnoses were used in the assignment of cardiovascular admissions as the organization of the Inpatient Registry does not allow discrimination between actual cardiovascular events and chronic comorbidities among secondary diagnoses. The diagnoses, according to ICD 9 and ICD 10, that were used in the definitions of cardiovascular and infectious admissions are listed in Additional file 1: Tables S1 and S2.

### Dialysis technique survival

Dialysis technique survival was compared between HHD and PD after day 90. Technique failure was defined as a change to another dialysis modality. Censoring was performed at dates of renal transplantation, recovered native renal function, death and the end of study, December 31st 2013.

### Statistical analysis

Assignment of Charlson comorbidity Index was performed with STATA software version 12. Determination of eligibility and matching were performed with SAS. All statistical analyses were made using IBM SPSS Version 23.

Kaplan Meier estimate and Breslow test were used for analyses of time to admissions or death. Mann-Whitney test was used for comparisons of admission per year and days per year. As matching was not performed using renal diagnoses cox regression analysess regarding hospital admission and dialysis technique survival were performed including renal diagnoses.

Results are given as medians and interquartile ranges (IQR).

## Results

### Patient characteristics

Between 1991 and 2012 152 patients started HHD as initial RRT in Sweden, according to the criteria used in this study [12]. Matching generated cohorts comprising 608 patients on IHD and 456 on PD. The mean age was 50 years in all three groups and 82% were male. Most patients had a Charlson Comorbidity Index of 0 (62%). The cohorts were not matched with respect to renal disease; the most common renal diagnosis was glomerulonephritis in all three groups followed by adult polycystic kidney disease for HHD and diabetic nephropathy for IHD and PD patients (Additional file 1: Table S3).

### Renal replacement therapies

Median follow up differed because of differences in survival and was 10.4 years for HHD, 7.0 years for IHD and 7.5 years for PD. Most patients changed RRT during follow up. The first period with HHD, IHD or PD was 2.1, 2.3 and 1.4 years, respectively. The most common shift of RRT modality was to renal transplantation. During follow up, 75% of the HHD patients, 68% of the PD patients and 51% of the IHD patients received 1 to 3 renal transplants (Additional file 1: Table S4).

### All admissions

**Fig. 1:**
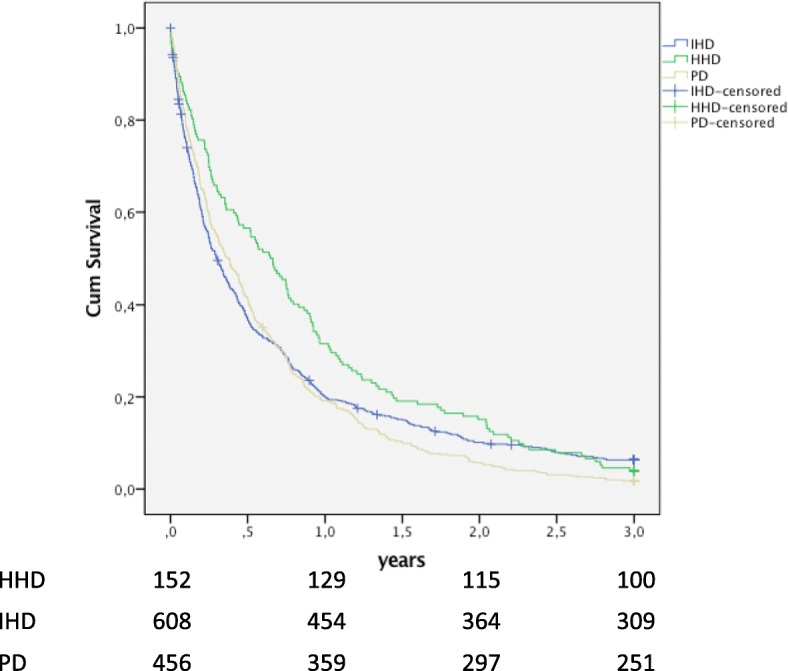
Time to first admission. Time to first all-cause admission during overall follow up (intention to treat) for patients with HHD (*n* = 152) as initial RRT compared with matched patients with IHD (*n* = 608; *p* < 0.001) and PD (*n* = 456; *p* = 0.001) as initial RRT

Patients on HHD had a significantly lower annual admission rate, 1.7, compared with IHD with 2.2 and PD with 2.8. The number of days in hospital was also significantly lower for patients on HHD, 12, compared with 14 with IHD and 20 with PD. During their initial RRT, 7% of the patients on HHD, 6% of the patients on IHD and 3% of the patients on PD had no hospital admissions (Table 2). Patients on HHD had significantly longer time to first admission, the median was 0.7 years compared with 0.3 years for IHD and 0.4 years for patients on PD. (Fig. 1).

**Table 2:** Admissions during initial HHD/IHD/PD treatment only and during overall follow up

	HHD	IHD	PD	HHD/IHD p value	HHD/PD *p* value
During initial HHD/IHD/PD treatment only (per protocol)					
Patients % *(n)*	93% (141)	94% (573)	97% (444)	–	–
Median annual admission rate [IQR] *n*	1.7 [0.9–2.8]	2.2 [1.1–4.4]	2.8 [1.3–5.3]	< 0.001	< 0.001
Median days per year [IQR] *n*	12.1 [6.6–21.4]	14.3 [6.4–33.3]	20.3 [9.3–41.2]	< 0.001	< 0.001
Median time to admission [IQR] years	0.7 [0.2–1.2]	0.3 [0.1–0.8]	0.4 [0.1–0.9]	< 0.001	0.003
During overall follow up (intention to treat)					
Patients % *(n)*	97% (147)	96% (583)	99,6% (454)	–	–
Median annual admission rate [IQR] *n*	1.3 [0.6–2.4]	1.6 [0.8–3.0]	1.5 [0.8–3.2]	0.014	0.023
Median days per year [IQR] *n*	6.5 [2.6–14.8]	8.5 [3.3–19.3]	8.9 [3.8–26.6]	0.048	0.001
Median time to admission [IQR] years	0.7 [0.2–1.2]	0.3 [0.1–0.8]	0.4 [0.1–0.8]	< 0.001	0.001

These differences in health care utilization persisted, when follow up time after changes to other RRT was included, as shown in the intention to treat analyses. Patients on HHD had a significantly lower annual admission rate, fewer days in hospital and significantly longer time to first admission compared with IHD and PD (Table 2). The time to first admission remained significantly longer for patients on HHD compared with IHD (*p* = 0.008) and PD (*p* < 0.001) after adjustment for renal diagnoses.

### Admissions with cardiovascular and infectious disease diagnoses

Cardiovascular diagnoses accounted for 14% of all hospital admissions during the period of initial RRT while 24% were due to infections diagnoses, when applying our definitions.

The majority of patients in all three cohorts had no admission due to a cardiovascular disease during their initial RRT. However, patients on HHD had significantly lower annual admission rate (HHD median 0 IQR 0–0; IHD 0 IQR 0–4; *p* = 0.002) and longer time to first admission (HHD 6.1 years; IHD 4.8 years; *p* = 0.017) compared with IHD patients (Table 3). The significant advantage for HHD in the annual hospital admission rate did not persist in the intention to treat analyses. For patients on HHD and PD there were no significant differences regarding admissions with a cardiovascular diagnosis.

**Table 3:** Admissions with cardiovascular diagnoses during initial RRT and during overall follow up

	HHD	IHD	PD	HHD/IHD p value	HHD/PD *p* value
During initial HHD/IHD/PD treatment only (per protocol)					
Patients % (n)	22% (34)	37% (225)	23% (103)	–	–
Median annual admission rate [IQR] n	0 [0–0]	0 [0–0.4]	0 [0–0]	0.002	0.711
Median time to admission [IQR] years	6.1 (2.7-NA)	4.1 [1.3–10.4]	4.1 (1.7–6.2)	0.009	0.296
During overall follow up (intention to treat)					
Patients % (n)	55% (83)	52% (315)	53% (244)	–	–
Median annual admission rate [IQR] n	0.06 [0–0.3]	0.07 [0–0.4]	0.07 [0–0.4]	0.512	0.724.
Median time to admission [IQR] years	10.0 [2.7–19.2]	6.8 [1.6-N.A]	7.6 [2.1–18.5]	0.032	0.269

Regarding admissions with infections, patients on HHD had a significantly longer time to first admission compared with IHD (HHD 3.4 years; IHD 2.8 years; *p* = 0.049) with follow up per protocol, but there were no significant differences in the annual frequency or the number of days admitted. Between HHD and IHD. In comparison with PD, patients with HHD had a significant advantage as to annual admission rate (HHD median 0 IQR 0–0.5; PD 0.3 IQR 0–1.5: *p* < 0.001) and time to first admission (HHD 3.4 years; PD 1.3 years; *p* < 0.001) with follow up per protocol. These differences persisted, though diminished, in the intention to treat analysis (Table 4).

**Table 4:** Admissions with infectious disease diagnoses during initial RRT and during overall follow up

	HHD	IHD	PD	HHD/IHD p value	HHD/PD *p* value
During initial HHD/IHD/PD treatment only (per protocol)					
Patients % *(n)*	46% (55)	43% (260)	53% (241)	–	–
Median annual admission rate [IQR] *n*	0 [0–0.5]	0 [0–0.6]	0.3 [0–1.5]	0.164	< 0.001
Median time to admission [IQR] years	3.4 [1.3–6.7]	2.8 [1.0–7.4]	1.3 [0.5–2.6]	0.049	< 0.001
During overall follow up (intention to treat)					
Patients % *(n)*	81% (123)	71% (432)	82% (373)	–	–
Median annual admission rate [IQR] *n*	0.3 [0.1–0.7]	0.3 [0–0.7]	0.4 [0.1–1.0]	0.435	0.047
Median time to admission [IQR] years	3.5 [1.3–7.3]	2.5 [1.0–8.8]	1.3 [0.5–3.9]	0.115	< 0.001

### Technique survival for HHD and PD patients

**Fig. 2:**
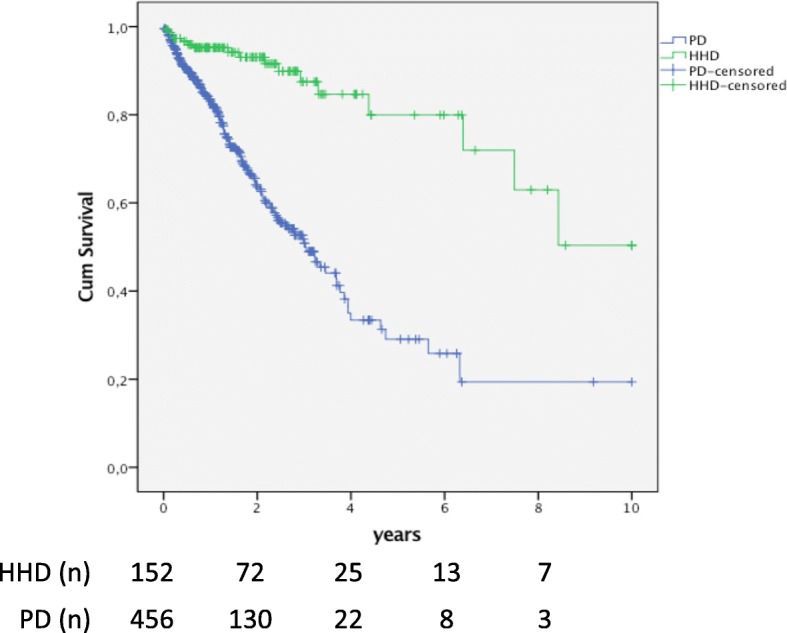
Technique survival for HHD patients and matched PD patients. Patients with HHD as initial RRT have an improved technique survival compared to patients with PD as initial RRT (*p* < 0.001). In this analysis censoring was performed at dates of renal transplantation, the end of study and dates of death. Only changes to other dialysis modalities were defined as events

Technique survival was compared after censoring for death and renal transplantation; it was significantly longer for patients on HHD compared with PD (*p* < 0.001; Fig. 2). Median technique survival was 10.0 (IQR 6.4–not available) years for patients on HHD and 3.0 (range 1.3–6.3) years for PD. Two- and five-years’ technique survival was 93 and 80% for HHD and 64 and 29% for PD, respectively. During follow up, 18 patients on HHD (12%) changed to IHD and 151 (33%) patients on PD, changed to IHD and one to HHD. Technique survival remained significantly longer for patients on HHD after adjustment for renal diagnoses (*p* value < 0.001).

## Discussion

This study shows reduced health care utilization for patients choosing HHD as their initial RRT compared with patients on IHD and PD. Hospital admission rate was 65% higher for patients on IHD and 33% higher for patients on PD compared with HHD. The number of days in hospital was 68% higher for PD and 18% higher for IHD compared with HHD. Time to first admission was longer for patients with HHD, 0.7 years as compared with 0.4 years for PD and 0.3 years for IHD. Finally, technique survival was better for HHD than PD.

The advantage of HHD for all-cause admission rates compared with IHD, in the present study, are in line with a Swiss study which included patients commencing RRT between 1970 and1995 [6]. Of note is that these differences between HHD and IHD were not found in studies from the US, utilizing data mainly from the twenty-first century [13, 15]. The only previous studies comparing HHD and PD were also conducted in the US during the twenty-first century and did not show an advantage for HHD in analyses restricted to incident patients [5, 14, 15]. The selection of patients to a dialysis modality might differ between Europe and the US with disparities in comorbidities, age and socioeconomic factors, the organisation of health care praxis and variations in the prescriptions of dialysis, all contributing to the observed discrepancies.

The European part of the DOPPS study reported a hospital admission rate of around one for IHD between 1998 to 2000 compared with 1.7 to 2.8 in our study for all dialysis modalities [18]. The USRDS reported an annual admission rate for all dialysis patients of 2.1 during 2005 which decreased to 1.7 for IHD and 1.6 for PD during 2014. A number of recent studies from the US, comparing HHD with IHD or PD during the twenty-first century, reported admission rates ranging from 0.7–1.8 for HHD; 1.1–1.7 for IHD and 0.7–1.9 for PD, all of which were lower than those found in the present study [5, 13–15]. These differences prevail for the number of days of hospital care per year, with lower numbers in other studies.

Thus, our results differ compared with other studies as well as in comparison with the European DOPPS and the USRDS, irrespective of dialysis modality, both in regard to differences in actual number of hospital admissions per year and number of days in hospital. In the present study, patients were included from 1991, which is earlier than any other study. During 1991 to 2000 the overall prognosis for dialysis patients was worse, both in Sweden and internationally, than from 2000 onwards [1, 2]. Moreover, during the nineties, Sweden had more hospital beds per capita than the US and the other European countries in the DOPPS study (https://data.oecd.org/healtheqt/hospital-beds.htm), which might have resulted in more frequent and longer admissions. There might also be methodological differences, as in the Swedish Inpatient Registry a new admission might be generated each time a patient is transferred to a new in-patient department during the same hospital stay.

Cardiovascular disease and infections are the most common causes of death in patients on dialysis [1, 2]. In our study, patients on HHD had significantly fewer admissions with a cardiovascular diagnosis compared with IHD patients. This is in line with other studies, which reported advantages for HHD regarding admissions with a cardiovascular diagnosis in comparison to both prevalent and incident IHD [5, 15]. Others have shown an advantage when comparing HHD with prevalent, but not with incident PD patients [5, 15]. Regarding admissions with infections, we registered significantly fewer admissions for HHD patients compared with PD. Earlier studies have, in accordance with our results, reported an advantage for HHD compared with PD, but contrary to our results, a disadvantage for HHD compared with IHD [5, 13].

Although patients in this study were matched for comorbidity at start of RRT, progress of and subsequent development of comorbidity, was probably lower in patients with HHD, and most likely contributed to the lower utilization of health care. This is supported by a better survival for HHD patients, which has previously been reported both by our group and others [10, 12]. Several studies have shown that the higher dialysis doses [19–22] and extensive patient education, [23–25], which are associated with HHD, are related to better fluid balance and phosphate control, both important factors in the development of cardiovascular morbidity [26–29]. In the present study there was no significant advantage for HHD concerning hospital admission rates with a cardiovascular diagnosis in the intention to treat analyses compared with IHD or PD. A weakened impact of the initial RRT after renal transplantation, could explain this absence of a significant carry over effect on the admission rates. In the present study, admission rates with cardiovascular diagnoses were low, 0.02–0.06 per year, compared with 0.36–0.48 with follow up according to intention to treat in the studies by Weinhandl [5, 13]. The patients in his studies were somewhat older, had higher proportions of diabetes as renal diagnoses and were not incident to RRT, factors that could well explain some of the difference in morbidity due to cardiovascular disease.

Another cause for this discrepancy might be due to how cardiovascular events are registered in the Swedish Inpatient Registry. The organization of the Swedish Inpatient Registry makes it impossible to discriminate between a cardiovascular event occurring during a hospital admission and a chronic cardiovascular comorbidity, which the patient had prior to admission and which has no direct impact on the cause of admission. For a chronic cardiovascular comorbidity to have an impact on the cause of hospital admission, it must be assigned the position of principal diagnosis. Thus, most cardiovascular ICD codes. Registered as secondary diagnoses, could not be used when classifying cardiovascular admissions, which most probably results in an underestimation of the number of admissions with a cardiovascular disease in the present study.

A possible explanation for the lower admission rate in HHD as compared with PD could be related to the resilience of the treatment modality. In accordance with earlier studies, the technique survival, was superior for HHD compared with PD [5, 30, 31]. In the present study, the 2 years technique survival was 93% for HHD and 64% for PD. Other studies from the US, Australia, New Zealand and Europe, have reported a two-year technique survival ranging from 75 to 96% for incident HHD patients and 64 to 74% for incident PD patients. Some of the differences between studies are related to methods and dialysis prescriptions. In one study from Canada, reporting a higher HHD technique survival, all patients had nocturnal HHD and some patients were completely dependent on caregivers for HHD treatment. In Sweden HHD is always self-care and administered by the patient in their own home. Possibly the setup with HHD administered by caregivers enabled a longer technique survival [32]. In another study, in which all patients used a single low-dialysate flow dialysis device, the reported HHD technique survival was lower compared with our study [5].

There are limitations to the present study, mainly due to the retrospective design. Despite strict matching and statistical adjustment for renal diagnoses, there is still a risk of differences between groups, especially concerning socioeconomic factors and smoking. However, health care in Sweden is publicly funded and the praxis and access to different RRT are relatively homogenous for all citizens. It is important to point out, that the results from the present study cannot necessarily be extrapolated to patients with older age, more comorbidities, different socioeconomic status or who, for whatever reason, are unable to be compliant to an independent home-based dialysis regime or societies with other healthcare structures. However, our results showing a lower admission rate for cardiovascular disease in patients on HHD compared with IHD and for infectious diseases in HHD compared with PD strongly suggest an effect of the modality rather than only from patient selection. A modality effect is further supported by our finding that these differences decrease after transplantation as seen in the intention to treat analysis. In a previous single centre report, we have shown that patients on HHD have a better control of fluid balance and hypertension compared with IHD, which could contribute to the lower tendency for cardiovascular admissions [9].

This study also has important merits. The SRR contains data on all patients in RRT and is updated when patients change treatment modality. All renal units in Sweden report to the SRR. Moreover, it is compulsory for all the hospitals in the country to report to the Swedish in-patient registry. Recently, the accuracy of the reported diagnoses has been validated [33]. Thus, the close to complete coverage of these registries enabled us to include virtually all Swedish patients starting HHD as initial RRT. This study also adds important knowledge compared with other recent studies, that are solely from the US, as it adds an European perspective and reflects effects of the different dialysis modalities in an entire and homogenous population with long-term follow up.

## Conclusions

In conclusion, this study provides new important evidence for patients when choosing their initial renal replacement therapy. In addition to better survival and higher likelihood of renal transplantation, patients on HHD spend less time in hospital as compared with patients on IHD and PD. This seems to be caused by decreased morbidity as HHD resulted in fewer hospital admissions with cardiovascular diagnoses compared with IHD and fewer admissions with infections than with PD. Moreover, HHD patients were more likely to be able to remain on the modality they had chosen. These advantages of HHD are strong incentives for promoting the use of HHD.

## Supplementary information

**Additional file 1:** **Table S1.** Cardiovascular diagnosis used in definitions of cardiovascular admissions. **Table S2.** Infectious diagnoses used in definitions of infectious admissions. **Table S3.** Patient characteristics at start of renal replacement therapy in a cohort of Swedish HHD patients and two matched control cohorts of IHD and PD patients. **Table S4.** Duration and frequency of initial and subsequent renal replacement therapies for cohorts of patients starting with HHD, IHD or PD.

## Data Availability

The datasets generated and/or analysed during the current study are available from the corresponding author on reasonable request.

## Abbreviations

GFR: glomerular filtration rate
HHD: home hemodialysis
IHD: institutional hemodialysis
PD: peritoneal dialysis
RRT: renal replacement therapy
SRR: Swedish Renal Registry
