# Supplementary material for: Fewer hospitalizations and prolonged technique survival with home hemodialysis– a matched cohort study from the Swedish Renal Registry
Source: BMC Nephrol. 2019 Dec 30;20:480. doi: 10.1186/s12882-019-1644-z (PMC6937632; doi:10.1186/s12882-019-1644-z)
Supplement: Supplementary file 1 — Additional file 1: Table S1. Cardiovascular diagnosis used in definitions of cardiovascular admissions. Table S2. Infectious diagnoses used in definitions of infectious admissions. Table S3. Patient characteristics at start of renal replacement therapy in a cohort of Swedish HHD patients and two matched control cohorts of IHD and PD patients. Table S4. Duration and frequency of initial and subsequent renal replacement therapies for cohorts of patients starting with HHD, IHD or PD. [file 12882_2019_1644_MOESM1_ESM.docx]

Table S1. Cardiovascular diagnosis used in definitions of cardiovascular admissions

| **ICD 10** | | | |
| --- | --- | --- | --- |
| Chapter | Included as principal diagnosis | Included as secondary diagnosis | Excluded |
| Diseases of the circulatory system (I) | All diagnoses, except those in the column excluded | I200, I21, I126, I60, I61, I62, I63, I64 | I02, I15, I78, I790, I791, I88, I89, I97, I98 |
| Other chapters | F01, G45, G46, G951, H34, K55, K763, K765, N280 | - | - |
| **ICD 9** | | | |
| Chapter | Included as principal diagnosis | Included as secondary diagnosis | Excluded |
|  | 390-459 | 410, 415, 430, 431, 432, 433, 434, 436 | 392, 398, 403, 404, 405, 425F, 429E, 443B, 446, 447, 455, 456, 457, 459A |
| Other chapters | 290E, 336B, 362D, 362W, 557, 573E | - | - |

Table S2. Infectious diagnoses used in definitions of infectious admissions

| **Chapter ICD-10** | **Included as principal or secondary diagnosis** |
| --- | --- |
| Certain infectious and parasitic diseases (A) | All diagnosis, except A50 |
| Certain infectious and parasitic diseases (B) | All diagnosis, except B90, B91, B92, B94 |
| Diseases of the nervous system (G) | G00, G01, G02, G042, G050, G051, G052, G06, G07 |
| Diseases of the eye and adnexa, and of the ear and mastoid process (H) | H600, H601, H603, H66, H700, H701, H702 |
| Diseases of the circulatory system (I) | I00, I01, I301, I320, I321, I33, I38, I39, I400, I410, I411, I412 |
| Diseases of the respiratory system (J) | J00, J01, J02, J03, J04, J05, J06, J10, J11, J12, J13, J14, J15, J16, J17, J18, J20, J21, J22, J32, J340, J350, J36, J390, J391, J40, J41, J42, J85, J86 |
| Diseases of the digestive system (K) | K102, K113, K122, K140, K20, K230, K35, K570, K572, K574, K578, K61, K630, K650, K659, K67, K750, K770, K800, K801, K803, K804, K810, K85, K901, K930, K931 |
| Diseases of the skin and subcutaneous tissue (L) | L01, L02, L04, L050, L08 |
| Consequences of external causes (T) | T 857, T 827 |
| Chapter ICD-9 | Included as principal or secondary diagnosis |
| Certain infectious and parasitic diseases | All diagnosis, 001-139  Except 090, 137, 138, 139 |
| Diseases of the nervous system (G) | 320, 321, 322, 323 (except 323H), 324 |
| Diseases of the eye and adnexa, and of the ear and mastoid process (H) | 380 (except 380E, 380F, 380W, 380X), 382 (except 382B, 382 C, 382 D), 383 (except 383W, 383 X) |
| Diseases of the circulatory system (I) | 390, 391, 420, 421, 422 |
| Diseases of the respiratory system (J) | 034, 373, 460, 461, 462, 463, 464, 465, 466, 473, 474A, 4741, 475, 478B, 478C, 478H, 478X, 480, 481, 482, 483, 484, 485, 486, 487, 490, 491, 510, 511A, 511B, 513, 530B, 680, 682 |
| Diseases of the digestive system (K) | 526E, 527D, 528A, 528B, 528C, 528D, 529A, 540, 562, 566, 567, 572A, 572B, 575A, 576B, 577A |
| Diseases of the skin and subcutaneous tissue (L) | 039, 680, 681, 682, 683, 684, 685, 686, 704W |
| Consequences of external causes (T) | 995W, 996G, 999D |

Table S3. Patient characteristics at start of renal replacement therapy in a cohort of Swedish HHD patients and two matched control cohorts of IHD and PD patients.

|  | **HHD** | **IHD** | **PD** |
| --- | --- | --- | --- |
| Patients *number* | 152 | 608 | 456 |
| Year of start *percent* *(n)*  1991-1999  2000-2009  2010-2012 | 52 % (79)  36 % (54)  13 % (19) | 48 % (290)  42 % (253)  11 % (65) | 48 % (221)  42 % (193)  9 % (42) |
| Median age  (IQR)*years* | 50.2  (42.1-58.2) | 50.1  (42.4-58.1) | 50.1  (42.2-58.0) |
| Gender *male percent (n)* | 82 %(124) | 82 % (496) | 82 % (372) |
| Charlson index *percent (n)*  0  1  2  3 | 63 % (95)  28 % (42)  8 % (12)  2 % (3) | 63 % (380)  28 % (168)  8 % (48)  2 % (12) | 63 % (285)  28 % (126)  8 % (36)  2 % (9) |
| Renal diagnosis *percent* *(n)*  Diabetes mellitus  Glomerulonephritis  Hypertension  APCKD^1^  Pyelonephritis  Other  Unspecified | 10 % (15)  30 % (46)  6 % (9)  15 % (23)  4 % (6)  28 % (43)  6 % (10) | 20 % (123)  25 % (149)  7 % (43)  10 % (62)  3 % (21)  23 % (138)  12 % (72) | 27 % (122)  28 % (126)  5 % (23)  9% (43)  3 % (12)  20 % (89)  9% (41) |

^1^Adult polycystic kidney disease

Table S4. Duration and frequency of initial and subsequent renal replacement therapies for cohorts of patients starting with HHD, IHD or PD

|  | **HHD** | **IHD** | **PD** |
| --- | --- | --- | --- |
| **Initial RRT**  **Median duration (IQR) *years*** | | | |
| First period with  HHD/IHD/PD | 2.1  (1.1-3.1; n=152) | 2.3  (1.1-3.9; n=608) | 1.4  (0.8-2.4; n=456) |
| Total treatment with  HHD/IHD/PD | 2.4  (1.2-3.6; n=152) | 2.6  (1.3-4.9; n=608) | 1.5  (0.9-2.7; n=456) |
| **Other RRT**  **Median duration (IQR) *years*** | | | |
| Renal transplantation | 8.9  (5.1-13.5; n=114) | 8.6  (3.8-12.3; n=312) | 8.4  (4.3-13.1; n=311) |
| HHD | - | 3.2 (2.3-6.8; n=10) | 0.8 (0.3-0.8; n=5) |
| IHD | 2.3  (0.6-4.8; n=36) | - | 3.0  (0.7-8.8; n=174) |
| PD | 0 | 1.7(0.6-2.7; n=15) | - |
